# Contemporary HIV/AIDS research: Insights from knowledge management theory

**DOI:** 10.1080/17290376.2017.1375426

**Published:** 2017-09-18

**Authors:** Chris William Callaghan

**Affiliations:** ^a^ PhD, Associate Professor in the School of Economic and Business Sciences, University of the Witwatersrand, Johannesburg, South Africa

**Keywords:** HIV/AIDS research, methodology, collaborative research problem-solving, real time research, theory development, Recherche sur le VIH/SIDA, collaboration de recherche et procedure de resolution, recherche en temps réel, théorie du développement

## Abstract

Knowledge management as a field is concerned with the management of knowledge, including the management of knowledge in research processes. Knowledge management theory has the potential to support research into problems such as HIV, antibiotic resistance and others, particularly in terms of aspects of scientific research related to the contribution of social science. To date, however, these challenges remain with us, and theoretical contributions that can complement natural science efforts to eradicate these problems are needed. This paper seeks to offer a theoretical contribution grounded in Kuhn’s paradigm theory of innovation, and in the argument by Lakatos that scientific research can be fundamentally non-innovative, which suggests that social science aspects of knowledge creation may hold the key to more effective biomedical innovation. Given the consequences of ongoing and emerging global crises, and the failure of knowledge systems of scientific research to solve such problems outright, this paper provides a review of theory and literature arguing for a new paradigm in scientific research, based on the development of global systems to maximise research collaborations. A global systems approach effectively includes social science theory development as an important complement to the natural sciences research process. Arguably, information technology and social media technology have developed to the point at which solutions to knowledge aggregation challenges can enable solutions to knowledge problems on a scale hitherto unimaginable. Expert and non-expert crowdsourced inputs can enable problem-solving through exponentially increasing problem-solving inputs, using the ‘crowd,’ thereby increasing collaborations dramatically. It is argued that these developments herald a new era of participatory research, or a democratisation of research, which offers new hope for solving global social problems. This paper seeks to contribute to this end, and to the recognition of the important role of social theory in the scientific research process.

## Résumé

La gestion du savoir en tant que domaine est un ensemble, qui inclut la gestion des processus de recherche dans ledit domaine. La théorie de la gestion du savoir peut potentiellement être utilisée comme support à la recherche concernant les problèmes comme le VIH, la résistance aux antibiotiques et autres. Particulièrement en termes de recherche scientifique liée à la science sociale. À ce jour, malgré l’avancé de la science et des recherches, ces problèmes persistent, et la contribution théorique qui peut servir de complément aux efforts des sciences naturelles pour éradiquer ces problèmes est insuffisante. Cette étude a pour but d’offrir une contribution théorique basée sur la théorie de paradigme d’innovation de Kuhn et le propos de Lakatos que la recherche scientifique peut être fondamentalement non-innovatrice. Cela suggère que des aspects de la production du savoir dans le domaine des sciences sociales peuvent être la clé d’une innovation biomédicale efficace. Vu les conséquences progressives des crises mondiales émergeantes, et l’échec des systèmes de savoir des recherches scientifiques à résoudre ces problèmes, cette étude fournit une revue de la théorie et de la littérature qui soutiennent un nouveau paradigme dans la recherche scientifique, basé sur le développement des systèmes globaux pour maximiser et les collaborations scientifiques. Effectivement, l’approche de systèmes globaux doit nécessairement inclure la théorie du développement des sciences sociales, ce qui est complémentaire aux processus de recherche des sciences naturelles. Mais, la technologie de l’information et la technologie des réseaux sociaux sont déjà développées au point où elles peuvent résoudre des problèmes du savoir à une échelle surprenante. Il est souvent accepté que ces développements engendrent une nouvelle ère de recherche participative ou une démocratisation de recherche, qui offre de nouveaux espoirs pour la résolution des problèmes sociaux au plan mondiale. Cette étude a pour but de contribuer de cette façon, et reconnait le rôle important de la théorie sociale dans le processus de recherche scientifique.

## 1. Introduction

Literature stresses the importance of social factors in HIV/AIDS epidemics, and the potential impact of emerging biomedicine ‘and its attendant opportunities and (perhaps unintended) social consequences’ (Friedman, Kippax, Phaswana-Mafuya, Rossi, & Newman, 2006, p. 959). Although an extensive literature exists on progress toward visions of ultimate eradication (see Burman, Aphane, & Delobelle, 2016), challenges such as ongoing societal stigma (Gilbert, 2016), poor infrastructure and treatment delivery issues (Koto & Maharaj, 2016) and continued risk-taking behaviours (Ngidi, Moyo, Zulu, Adam, & Krishna, 2016) persist. This article makes the argument that certain recent developments in knowledge management enabled by technological advances offer hope for the ultimate eradication of HIV/AIDS, and offers insights into how these novel developments might contribute to this end. HIV/AIDS, however, is not alone among a host of global disease threats. Rapidly spreading antibiotic resistance heralds a post-antibiotic era (Gallagher, 2015), or antibiotic apocalypse (Ash, 1996); a world in which childbirth, even minor surgery, minor injuries and exposure to tuberculosis or other bacterial infections can be life-threatening. Similarly, climate change has been discussed in apocalyptic terms, or as a phenomena that, if not addressed, can result in large-scale loss of life (Feinberg & Willer, 2011), and HIV antiretroviral resistance (Herman, 2016) might also have catastrophic consequences if it follows the same trend as that associated with antibiotic resistance. Knowledge management has to date, however, failed to offer outright solutions to these problems. This paper argues, however, that in light of recent technological advances, knowledge management and social sciences theory development can now offer an important complement to the ‘natural’ sciences processes of scientific research, with important implications for research problem-solving.

Certain reasons for the inadequate responses to these emerging threats have been mooted. It is possible that given exposure to knowledge of threats on an ‘apocalyptic’ level, or potentially catastrophic global threats, individuals may deny or discount their existence, exhibiting decreased willingness to address them (Feinberg & Willer, 2011). There are other reasons for failure to address apocalyptic threats, and many other crises (not to mention global conflict) which might qualify for apocalyptic status, or have high potential human life costs. Whereas denial (Feinberg & Willer, 2011) might be one form of response to such imminent and serious threats, it is argued in this paper that another more useful response to these challenges is emerging, according to different literatures.

This response is broadly categorised here as a trend in research and collaborative problem-solving termed probabilistic innovation, or innovative problem-solving processes which seek to dramatically increase the probability of solving problems through harnessing economies of scale in knowledge management. The key characteristic of this body of literature can be described as a focus on dramatically increasing the global intensity of *collaborations* between problem-solvers. What is novel, or new, about these movements is that they share a common characteristic, namely arguments for the importance of social science theory as a complement to natural science methods of scientific research. Further, this literature suggests bottlenecks to innovative research capacity can also have their roots in human behaviour and systems of researchers.

Following Kuhn’s (1962) arguments that paradigms change only as the shared values and norms of researchers change places social science at the heart of constraints to innovation in scientific research, rather than the reductionist logics which frame scientific progress as a function of purely natural science theory. Certain of the highest cited papers and most influential academic books were first rejected by journal reviewers and editors, including Nobel Prize winning manuscripts in Physics, Chemistry, Physiology and Medicine, raising ‘important questions about current publishing policies which govern the dissemination of new information’ (Campanario, 2009, p. 558). In relation to the failure of Newtonian theory to integrate new theory development into its core, Lakatos (1970) argues that scientific progress can be constrained by the failure of theoretical frameworks to be responsive to new ideas, and that the structure of academic processes can hold back innovation.

Further, ‘academic market failures’ in academic research are commonplace (Dewald, Thursby, & Anderson, 1986), as constraints to innovative research and to the progress of science itself exist within the processes by which academic research is published and disseminated (Bornmann, 2010, 2011). Further, ‘critics often argue that peer review operates to regulate paradigmatic science (in the Kuhnian sense) rather than to welcome brand new knowledge’ and there is therefore a ‘real risk that evidence contrary to the established views can be suppressed or discarded’ (Campanario, 2009, p. 559). Empirical research in certain instances has been found to be associated with biases (Henrion & Fischhoff, 1986), including ‘file drawer bias’ where studies failing to reject the null hypothesis literally remain in ‘file drawers’ (De Long & Lang, 1992) and serious concern has long existed across many scientific disciplines about the processes by which science is disseminated, in that theoretical innovation can be constrained by gatekeepers (Peters & Ceci, 1982). Notwithstanding bias in theory testing methods, dissemination of theory for fair criticism and contestation is perhaps a necessary condition for the progression of science.

Longstanding evidence suggests social effects in the academic publication system can constrain innovative idea generation. Mahoney (1977, p. 161) documents a ‘tendency for humans to seek out, attend to, and sometime embellish experiences that support or “confirm” their beliefs,’ which has led to a bias against the dissemination of new perspectives, which is fundamentally at odds with Popper’s (1972) notions of falsification, which require theoretical ideas to disseminate prior to their testing, or to provide opportunities for empirical falsification. Serious issues related to the resistance of academics themselves to scientific discovery Campanario (2009) seem to support Kuhn’s (1962) and Lakatos’s (1970) seminal arguments that constraints to scientific innovation can primarily have roots in human behaviour, and that social science has an important role in addressing these problems. Campanario (2009, pp. 550, 560) argues that the ‘important topic of scientists’ resistance to scientific discovery’ is under-researched, as case studies show that ‘scientists with something truly original to communicate often have to fight against the silence, the lack of interest, and as a result the absence of citations and recognition.’

This research therefore seeks to identify and articulate a stream of literature which argues a new paradigm in research problem-solving is emerging, on the back of emergent technologies which enable new opportunities, and that this emerging body of theory has important implications for HIV research as it places social science research as an important complement to natural science in problem-solving. The theoretical contribution of this article is therefore in its identification and delineation of boundary conditions to research problem-solving theory. Clear and practical examples are also offered to support the arguments made here, and the paper also makes a contribution in offering a review with important insights for practitioners involved in different aspects of HIV research.

It is therefore argued here that this movement to maximise collaborative engagement between stakeholders, members of the problem-solving crowd and all manner of those affected by a problem effectively represents a paradigm shift in problem-solving, and that social media and technological advances, including big data Manyika et al. (2011) have enabled a potential for problem-solving hitherto relatively untapped. The big data era has increased the transparency and usability of information (Manyika et al., 2011), and offers the potential for fundamental disruption in science and particularly in biological discovery (Swan, 2013). There is perhaps hope of a curative paradigm in science (outright cures for disease, albeit less profitable for pharma firms) and hope of advances in social science that can solve problems like global warming at the source, or in terms of human behaviour.

However, within a scientific context of dramatically increased information and data proliferation, as ‘the breakneck pace of genome-technology development has revolutionised bioscience research’ (Hayden, 2014, p. 294) and information accumulation rates exceed predictions of Moore’s Law, transmission to problem-solving outcomes is lacking (from big data to ‘big’ knowledge); the missing portion of the contribution of big data to global problem-solving equation may be what humans uniquely contribute, or complex and tacit (Nonaka, 1994) problem-solving knowledge creation. It is argued in this paper that developing a stream of literature, or a field, explicitly focused on maximising human collaborations (as a dimension of knowledge management) could act as a counterpoint to the rapidly burgeoning field of big data and information management and enable a curative paradigm in health as well as more effective problem-solving in social science. This paper seeks to identify and link literature in support of this objective, and to make an argument that this literature may usefully be synthesised under the banner of an emerging field, namely probabilistic innovation, driven by a social movement toward the democratisation of knowledge (and therefore science, medicine and social science).

Probabilistic innovation theory, with its knowledge management ‘lens,’ focuses on the human dimension of problem-solving, predicting that exponential increases in problem-solving inputs can decisively increase probabilities of solving complex yet inherently ‘solvable’ problems (where the solution to such problems is realistically a function of problem-solving attention). However, the extent to which higher volumes of human problem-solving inputs transmit to problem-solving outputs is difficult to predict. The lack of knowledge of constraints to this transmission is associated with ongoing social costs. Costs of lack of knowledge typically accrue to those most powerless in society, or those most vulnerable to crises. This paper therefore argues that this knowledge problem (lack of knowledge of how to increase this transmission factor) is a critically important research question in all fields related to catastrophic social problems, for the following reasons.

*First*, development of theory that threads out constraints and principles related to maximising collaborative problem-solving, to the extent that knowledge creation increases can match information creation and the rise of big data, may be key to solving apocalyptic problems. The era of big data needs to be matched by a concurrent era of ‘big knowledge.’ One channel through which this might be achieved is enabling ‘big collaborations.’ Scale advantages associated with exponential increases in collaborations may increase probabilistic engagement with available data, and populate the problem space sufficiently. However, populating a relatively unpopulated problem space (big data) with human problem-solving inputs may require a radical approach. This paper argues that such a radical approach is possible, premised on principles related to the democratisation of knowledge. Use of the crowd, expert and non-expert crowdsourced research and problem-solving inputs, as well as a theoretical framework relating to proactively maximising collaboration through developing global systems, seems key to attaining this.

*Second*, to attain a ‘Moore’s Law’ scale increase in knowledge commensurate with big data increases, a radical approach to increasing collaborations is needed, one which problematises knowledge creation and looks to lessons from extreme cases to develop and synthesise streams of literature around the human dimension of accelerated real-time problem-solving. A sociotechnical infrastructure to operationalise collective intelligence of human problem-solvers and researchers might contribute to this end. Different perspectives on this are included in sections which follow.

*Third*, if the potential for collaborations is increased dramatically (and if problem-solving from expert and non-expert crowd is ultimately harnessed), congestion then becomes a (the) critical issue; congestion associated with very high volumes of problem-solving inputs would follow successful attempts to generate inputs. An analogy might illustrate this challenge. If an airport could land and take off about a hundred aircraft in a day, exponentially increasing those landing would be akin to increasing problem-solving inputs using a crowdsourced R&D platform, and exponentially increasing those taking off to problem-solving outputs. Such an airport would then need to manage congestion in such a way to be able to land and take off hundreds of thousands of aircraft in a day. Posed this way, such a research problem might seem insoluble, at least at the scale discussed here. This research problem, however, if solved, holds the promise of radical innovations in socially important problem-solving. Solving cancer, HIV/AIDS, diabetes, Ebola, antibiotic resistance, as well as a host of other problems might truly become a simple function of maximising problem-solving inputs, if these inputs could transmit to successful outputs. It is this logic this paper seeks to develop, with specific reference to literature related to how collaborative problem-solving can be maximised; with the ultimate aim to empower a social movement dedicated to an ‘extreme’ focus on problem-solving, or radical change based on the democratisation of science (Callaghan, 2015). Having provided an outline of the arguments of the paper, and a justification for its contribution, theory relating to scientific collaboration is now introduced.

## 2. Scientific collaboration at the extreme

At the heart of the quest for real-time research capability is the need for scientific collaboration. One may argue, however, that there is already collaboration between scientists across the world in support of HIV/AIDS research. Similarly, there are big institutions which also facilitate collaborations in support of global problem-solving, and the same exists for collaborations between schools, individual researchers and students. In order to more strongly outline the originality of this research, it is necessary first to highlight the limits to what is currently being done, and to offer a perspective of what scientific collaboration at the extreme may contribute to societal stakeholders. It is argued that two fundamental constraints exist to collaborations. These are considered as follows.

*First*, notwithstanding extensive collaboration, the seminal *knowledge aggregation problem* (Hayek, 1945; von Hippel, 1994) poses constraints to the collaborations of researchers. Given most of the expert knowledge required to solve scientific problems is tacit (Nonaka, 1994; Polanyi, 1973) and cannot therefore be separated from individuals, it is difficult to ‘aggregate’ this tacit knowledge across individuals. Geographic, temporal and other logistic challenges make tacit learning between individuals even more difficult. Arguably, this is a dominant constraint to solving problems in biomedicine, as it is simply not possible to get large numbers of people to work together and enable tacit expert knowledge interactions using ‘conventional’ methods of collaboration. It is argued here that expert crowdsourcing, or crowdsourced R&D, has yet to be taken up as a viable alternative to conventional collaborations. Naturally, the question arises – why crowdsourced R&D – and why is its use in curative disease research not yet widespread, notwithstanding its extensive use in data collection and analysis in medical research? Arguably, its use is already widespread, but limited to platforms which collect information for proprietary ends.

*Second*, another fundamental constraint to social research problem-solving therefore exists in the proprietary structure of crowdsourced R&D. For example, platforms like InnoCentive (InnoCentive, 2016) put biomedical and other scientific problems online for solving, in a process akin to open-source software development. The problem, however, is that unlike open-source software development, solutions become proprietary knowledge of the firm putting the problem on the site, and this knowledge is not released back into the crowd (Callaghan, 2014a). Arguably, this is one of the reasons why crowdsourced R&D has not delivered the successes open-source software development has, such as Android and Linux. Nonetheless, it is argued that certain opportunities for harnessing collective intelligence through maximisation of collaborations discussed in this article can offer important insights to those in HIV/AIDS research. Key to these opportunities is social theory development and the ability to take advantages of new developments in technology that can enhance such collaborations.

At its most extreme, radically enhanced collaborations may entail crowdsourced R&D eliciting high volume inputs from large numbers of problem-solvers from the crowd, expert and non-expert, generating millions or tens of millions of suggestions and inputs, and channelling these to generate useful high-volume problem-solving. In order to understand scientific collaboration at the extreme, it might be useful to understand instances of crises where collaborations have been successful, and work which has already called for the development of maximised collaborative systems.

According to Malone and Klein (2007), harnessing human collective intelligence to solve global societal problems (their specific example being global warming) requires global interconnectivity. A global trend toward increasing research collaboration is evident in literature over time. Adams, Black, Clemmons, and Stephan (2004) report an increasing trend in the size of scientific teams and institutional collaborations and an acceleration of the trend toward larger and more dispersed teams. It may be necessary to proactively develop a global open knowledge creation system to radically increase collaboration, and to eliminate geographical constraints to collaborations. Examples of an increased focus on enabling collaboration in HIV research include the cooperative Spanish HIV BioBank (Garcia-Merino et al., 2009), and the use of interactive web sites for histocompatibility (De Groot et al., 2009). Increasing opportunities for collaboration when combined with recent technological advances such as massively parallel sequencing methods (Bushman et al., 2008) offer important opportunities for HIV research, but geographic and other constraints to collaboration make study of how to use technology to improve collaborations a critical aspect of ongoing HIV research.

Technology has not only reduced geographical constraints to collaboration, but information technology has been found to be an ‘equalising force’ in academia, empowering those from non-elite institutions and reducing gender bias in academic research productivity as well as enabling collaborative opportunities (Ding, Levin, Stephan, & Winkler, 2010). Adams et al. (2004) acknowledge that ‘lagging public funding of scientific research’ might also compel universities to increasingly engage in institutional collaborations. Real-time research responses, however, require very large-scale collaborations which span boundaries, or ‘loose’ collaborations. An example of the success of such a response is how the threat of Severe Acute Respiratory Syndrome (SARS) was averted through a near real-time response.

Surowiecki (2004, p. 161) notes that the identification of the SARS virus was effective, despite a process based on loose collaborations; this, however, reflects how much of science is conducted:

Researchers, particularly experimental researchers, routinely work in large groups, and it’s no longer strange to see scientific papers that are co-authored by ten or twenty people. (This is in sharp contrast to the humanities, where single authorship remains the norm.) A classic example of this phenomenon was the discovery, in 1994, of the quantum particle called the ‘top quark.’ When the discovery was announced, it was credited to 450 different physicists.

The success of SARS research is an example of the success of informal collaborations. The successful management of serious crises in real-time offers lessons for probabilistic innovation approaches. Relating these examples to the extreme case of maximum collaboration, the question arises as to what is the underlying rationale behind increasing productivity through collaboration? Surowiecki (2004, p. 161) offers the following arguments for why scientists collaborate.

Why do scientists collaborate? Part of it is the result of what’s often called the ‘division of cognitive labour.’ As science has become ever-more specialised and as the number of subfields within each discipline has proliferated, it’s become difficult for a single person to know everything he needs to know. This is especially true on experimental science, where sophisticated machinery demands unique skills.

One might argue that a hyperspecialised model of research with proliferation of discipline specialisation has, while deepening knowledge stocks, also constrained our ability to respond innovatively to important social problems in the absence of a radical spur to action as in the case of the SARS response. A successful systematic attempt to build a probabilistic innovation platform may to some extent replicate the success of the SARS initiative in that large-scale scientific collaboration could be facilitated, increasing the chances of successful societal problem-solving.

## 3. Congestion theory

Given the division of cognitive labour advantages accruing to collaborative research, what then would be the primary limiting features of a model seeking to maximise scientific (expert and non-expert) collaborations in support of accelerated research problem-solving? A primary limit to maximising scientific collaboration is congestion. Study of motor vehicle traffic flows might offer insights relevant also to Internet traffic in general, and more specifically for the management of maximised crowdsourced R&D inputs. Problems of traffic flows are well researched; two different literature camps have emerged over time, namely physicists, arguing spontaneous ‘jams’ occur, akin to the movement of water or particles, and engineers, including traffic engineers who conceptualise jams to be caused by obstacles, which can therefore be managed, or removed (Surowiecki, 2004). Traffic jam congestion has been the subject of simulation research (Munoz & Daganzo, 2002; Nagel, 1994), and a body of literature exists which might be usefully applied to crowdsourced R&D applications in contexts of congestion. Modelling high volume Internet traffic relationships might offer useful insights into how maximum inputs into crowdsourced R&D processes can be optimally managed. If circularity in the use of the crowd is attained, such as crowd funding used to support crowdsourced R&D (Callaghan, 2014b) the growth of the system might become financially sustainable, which may increase knowledge inputs at the cost of congestion. The ‘airport’ analogy research problem highlights the nature of these constraints.

In the case of airport traffic, airport landing fees would need to reflect congestion costs, where charges for the use of alternative routes do not reflect congestion costs at the margin, congestion can be problematic; furthermore, charges also have an informational role, and offer capacity information (Vickrey, 1969). In large-scale crowdsourced data collection and analysis seeking to facilitate real time research, crowdsourced R&D inputs would therefore need to be differentiated according to their relative value to ensure overall costs of congestion from crowdsourced inputs are linked to benefits. Such a mobilisation of crowds of problem-solvers, expert or not, would require crowd management. Given the centrality of crowdsourced ideas and problem-solving to probabilistic innovation, the question arises as to what extent the crowd itself can solve the congestion problem, as the ultimate expression of collaboration. The crowd has taken on an increasing share of health-related research, in the wake of technological advances enabling connectivity. However, to be able to manage congestion associated with high-volume open knowledge creation, these processes need to be considered as systems.

## 4. Systems theory

Crowdsourced R&D collaborations could benefit from guidance provided by developing theory relating to systems, or systems analysis. Application of systems analysis in broader medical research is not new. As an example, systems analysis has been used to investigate adverse drug effects (Leape, Bates, Cullen, Cooper, & Demonaco, 1995). The rise of collaborative knowledge systems such as Wikipedia which applies technological tools and managerial dynamics to structure and manage its content; such systems are sociotechnical systems that enable collaboration not only between humans but between humans and automated content agents (Niederer & van Dijck, 2010). Understanding the complex dynamics of a system which seeks to maximise knowledge flows and process them to maximise outflows may benefit from incorporating a systems analysis approach. Design of large-scale knowledge creation systems can also draw useful insights from analysis of disasters.

System design based on systems theory can make error occurrence less likely, and can allow errors to be ‘absorbed’ or detected and corrected prior to harm; these derive from the systems theory of causation, which suggests a process whereby systems failures underlying errors are identified, and systems are then designed to improve performance (Leape et al., 1995). The design of a global knowledge creation system may support crowdsourced R&D (Callaghan, 2015), where constraints to maximum collaboration are transcended, drawing from a systems analysis approach. The inadequacies of human decision-making and interactions pose challenges, however, to any attempt to build a model of maximised collaborations.

The systems analysis approach considers all decision-making a function of modelling, offering mental models of decision-makers, which are, however, ‘fuzzy, incomplete, and imprecisely stated’ (Forrester (1971, pp. 4–5). Maximised collaborations are therefore also constrained by the inherent characteristics of human researchers. Human systems, however, have also arisen in the form of organisations, institutions and networks which face host of other barriers which constrain collaborations, posing further challenges which are specific to crowdsourced R&D. An example of crowdsourced R&D in the medical field is Ekins and Williams’s (2010, p. 393) vision of crowdsourced pharmaceutical research:

Living in our connected world, pharmaceutical researchers can communicate in a variety of ways to leverage ideas from around the globe. These ideas do not have to come from within the walls of a single organisation. Taking this further: why limit access to just ideas? Open tools and data could feed an ecosystem. They could also breed a new class of researcher without affiliation, who has allegiance to neither company nor research organisation. They test their hypotheses with data from elsewhere, they do their experiments through a network of collaborations, they have no physical lab; while a shared cause may not be essential, confidentiality agreements and software may unite them as a loose cooperative.

Open modes, however, can be ineffective if certain conditions are not met. It has to be possible to evaluate solution inputs at low cost; for software screening for bugs this can be quick and low cost but this is not the case when it would take expensive and time-intensive experimentation to test the worth of ideas; in some cases the evaluation burden cannot be easily shifted to customers (as in the case of Threadless), and in other instances, participation is not easy as problems cannot be partitioned into well-defined segments that can be worked on by different groups (Pisano & Verganti, 2008). Notwithstanding challenges facing open modes of innovation, open mode applications of crowdsourced R&D to drug discovery is dramatically on the rise, according to Ekins and Williams (2010, p. 393/394):

A recent example of the power of crowdsourcing is the availability of freely accessible online resources to enable and support drug discovery. For instance, online databases, including PubChem, Chemical Entities of Biological Interest on ChEBI database (http://www.ebi.ac.uk/chebi/), DrugBank (http://www.drugbank.ca/), the Human Metabolome Database (www.hmbd.ca) and ChemSpider (http://www.chem.spider.com). These represent either government of privately funded initiatives with vastly differing resources and scopes … Sometimes, there are synergistic benefits of crowdsourcing; for example, the efforts behind the ChemSpider platform, originally a hobby project housed from a basement and recently acquired by the Royal Society of Chemistry, has been acknowledged to have greatly enriched the content in the NIH’s PubChem.

These crowdsourcing systems offer a way to harness collective human intelligence. Malone and Klein (2007) stress harnessing human collective intelligence requires global interconnectivity that extends beyond simple discussion rooms, blogs and chat rooms; these are typically not robust to, for example, the influence of narrow topical issues or vocal participants. Conscious systems design might therefore be used to develop global connectivity that goes beyond social media, and model human shortcomings which constrain collaborative behaviour. Developing systems of interconnectivity to maximise collaboration may require an explicit systems approach, which explicitly models human shortcomings as part of systems. Forrester (1971, p. 110) argues that:

 … the human mind is not adapted to interpreting how social systems behave. Our social systems belong to the class called multi-loop nonlinear feedback systems. In the long history of evolution it has not been necessary for man to understand these systems until very recent historical times. Evolutionary processes have not given us the mental skill needed to properly interpret the dynamic behaviour of the systems of which we have now become a part.

The primary difference between mental models and system dynamics simulation models lies in the way the latter are ‘explicit about assumptions and how they interrelate’ as any concept ‘that can be clearly described in words can be incorporated in a computer model,’ a process which forces ideas to be clarified and unclear and hidden assumptions to be surfaced (Forrester, 1971, p. 5). Knowledge of systems theory is therefore considered to be particularly important in ensuring the diffusion of innovations, and to the development of global knowledge systems to enhance connectivity.

## 5. Global knowledge creation systems

A global knowledge creation system will require systems of innovations to spread both across organisational boundaries and within them. Intra-organisational implementations of innovations frequently fail, costing time and resources, potentially contributing to organisational failure; these measures rely on individual adoption decisions in a process mirroring how products or services innovations diffuse in a market (Wunderlich, Größler, Zimmermann, & Vennix, 2014). Human intuition, however, is shaped by feedback from simple systems, where causes of problems are proximal to their symptoms, but complex dynamic systems typically have causes which are separated from the problem both in time and space; what might appear to be causes can be coincident occurrences caused by feedback-loop dynamics of a larger system which also produce the problem itself (Forrester, 1971). Researchers acting as part of this larger system will need to have a holistic view of the system itself.

Developing a sociotechnical system to support a maximally collaborative research network requires the management of social systems, both within and across organisations. Social systems typically have sensitive influence points which can be used to change behaviour, but these are difficult to correctly locate; the implication of this is that policy efforts can be counterproductive, worsening a problem, and a further challenge exists in that policies found to be effective in the long run often tend to worsen the problem they seek to address in the short-run (Forrester, 1971). Shum et al. (2012) acknowledge that the development of a sociotechnical infrastructure is necessary in order to operationalise collective intelligence and to focus it to develop solutions to problems; this emerging field of literature builds on previous work that has sought to develop a conceptual framework relating to the improvement of human intellectual effectiveness, such as the work of Engelbart (1962). Key to systems approaches to research problem-solving is the notion of open systems of innovation.

There seems to be general agreement that open systems of innovation offer the potential for maximising collaborative knowledge creation, even within organisations. In totally open collaboration, or crowdsourcing, ‘everyone (suppliers, customers, designers, research institutions, inventors, students, hobbyists, and even competitors) can participate,’ giving rise to dramatic successes such as Linux, Apache and Mozilla (Pisano & Verganti, 2008, p. 78) as examples of open-source software engineering. In light of these successes, which rival closed innovation systems such as Microsoft and Explorer, the question is whether such success can (ultimately, given time and explicit focus) *be replicated in solving social problems like HIV, antibiotic resistance and the like using open-source crowdsourced R&D*, given the urgency associated with the potential for ‘apocalyptic’ consequences associated with these health threats. Arguably, the potential of open networks of problem-solving to solve these problems remains underexplored.

Closed networks, ‘in contrast, are like private clubs’ where ‘you tackle the problem with one or more parties that you select because you deem them to have capabilities and assets crucial to the sought-after innovation’ (Pisano & Verganti, 2008, p. 78). These bodies of theory offer a host of different strategies that can be used to maximise research collaboration both within and across organisational or national boundaries. But in order to radically accelerate problem-solving, a concerted effort to maximise collaboration is needed, on the scale that it can itself be considered a radical process innovation. Sustaining these changes, however, is also expected to in turn generate a host of other challenges, which will also require attention, not least of which is resistance to new ideas or even the rejection of innovations after adoption.

Decisions to reject an innovation after adoption have been likened in the literature to disease susceptibility post recovery; communication underlies innovation diffusion, and the concrete structure of communication reveals which groups interact with each other (Wunderlich et al., 2014). Engendering maximum collaboration requires a match between the ideal and the realities of organisational dynamics. In certain instances, closed structures persist. According to Pisano and Verganti (2008, p. 79), adopting closed structures represents an implicit bet that (i) the knowledge domain from which the solution to the problem is derived has been chosen correctly, and (ii) that collaborators chosen are best in the field; whereas the advantage of open networks is primarily in their potential to attract ‘an extremely large number of problem solvers and, consequently, a vast number of ideas’ as well as it being not necessary to identify best knowledge domains nor the most appropriate experts in these domains. Populating the ‘coalface’ of a problem landscape of global societal problem with very large numbers of human problem-solvers, who then respond to the unique configuration of the problem at each point of contact, necessarily requires support mechanisms, and some degree of quality control as well as appropriate incentives (Callaghan, 2015).

## 6. Innovation contests and global systems

Innovations are increasingly enabled through private or public funding, and also through novel incentives, such as those provided by crowdsourcing X-prize type awards (http://www.xprize.org/future-x-prizes/life-sciences) (Ekins & Williams, 2010). Innovation contests can be a useful way to incentivise the effort and inputs required to maintain a high-volume problem-solving system. Ekins and Williams (2010, p. 394) stress that such systems require

some degree of focus initially to such an open drug discovery model to increase the probability of success, maybe around a neglected disease like Malaria or Tuberculosis (TB), or even rapidly emerging diseases (like swine flu), to demonstrate that it is more than a utopian concept.

Arguably, such research has become a necessity, and failure to maximise collaborative efforts in this way may entail ongoing human costs.

Having considered different conditions dictating different approaches to high collaboration knowledge creation, the question remains as to what commonalities underlie crowdsourced R&D applications across different problem domains. Despite much variance in R&D mechanics across industries, particularly in terms of cost structures, success rates and market rewards, the innovation process ‘is remarkably similar across industries’ as drug candidates ‘in a pharmaceutical development process, TV shows in an entertainment company, and proposals in a venture capital firm all flow through a conceptually similar innovation process,’ which starts with generating candidate opportunities and filtering those most promising (Terwiesch & Xu, 2008).

A certain degree of flexibility in the problem-solving process is another advantage associated with the use of crowdsourced R&D in a system of open maximised collaboration. Pisano and Verganti (2008, p. 79) explain this further as follows:

With open participation, you don’t need to know your contributors. Indeed, the fact that you don’t know them can be particularly valuable; interesting innovative solutions can come from people or organisations you might never have imagined had something to contribute. That is the concept behind Threadless.com, a largely online retailer of T-shirts, whose designs come from the masses. By operating an innovation mall where 600,000 members submit proposals for about 800 new designs weekly, Threadless gets a steady flow of unusual and singular ideas. (Mall members and visitors to the website vote on the designs, but the Threadless staff makes the final decision on which ones to produce and rewards their creators).

For Shum et al. (2012, p. 109), the development of, and application of, collective intelligence is an urgent imperative to address the ‘urgent, systemic problems now threatening the sustainability of societies.’ Key to this, according to Shum et al. (2012), is the development of a global sociotechnical infrastructure, an example of which is the Global Participatory Platform (GPP). Shum et al. (2012, p. 109) conceptualise this type of sociotechnical system, or GPP as:

a framework for different stakeholders to find their ecological niches at different levels within the system, serving the functions of (i) sensing the environment in order to pool data, (ii) mining the resulting data for patterns in order to model the past/present/future, and (iii) sharing and contesting possible interpretations of what those models might mean, and in a policy context, possible decisions … a resilient, epistemic ecosystem, whose design will make it capable of self-organisation and adaptation to a dynamic environment, and whose structure and contributions are themselves networks of stakeholders, challenges, issues, ideas and arguments whose structure and dynamics can be modelled and analysed.

In the workplace, new forms of intermediaries have sprung up to support the distribution of tasks and subordinate tasks to the crowd; these include Amazon’s Mechanical Turk (small tasks requiring human skills), TopCoder (programming and design and development competitions), Guru.com (talent searchers in skill-based serives), eLance (graphic design, computer programming and web development), LiveOps (call centre services), Innocentive (competitions in solving science and technology problems), oDesk (services), CastingWords (transcription services), Crowdflower (verifying information and sorting images) and Samasource (distributing computer work to those in disadvantaged regions of the world) (Malone, Laubacher, & Johns, 2011).

Jobs that used to be undertaken by one person are now done by many in the crowd, a process termed ‘hyperspecialisation’ (Malone et al., 2011). This process echoes the argument made in this paper, that the crowd holds the key to the acceleration of knowledge creation, as complex problems represent a large problem space, and real-time problem-solving might be better enabled through populating the problem space with large numbers of ‘solvers.’ Hyperspecialisation, and the way it is unfolding in the workplace, might offer unique insights that can be applied in the quest for real time research. While these changes offer proof of concept of how work can fit with high-volume crowdsourced systems (and that very large economies of scale are possible), these developments may also provide the basis for a synthesis of crowd-specific work methods with developing networks of large-scale collaborations.

The proliferation of the large-scale open innovation networks is a feature of the current global problem-solving landscape. Ekins and Williams (2010, p. 393) stress that this vision has already attained fruition, and applications are now widespread:

Such approaches may become more commonplace, like the Open Innovation efforts represented by companies such as NineSigma (http://en.wikipedia.org/wiki/Ninesigma) and Innocentive (http://en.wikipedia.org/wiki/Innocentive). The One Billion Minds approach for open innovation (http://www.onebillionminds.com/) has already been mapped into the Life Sciences, where a million minds in the community have been called to participate in community annotation in Wikeproteins (http://genomebiology.com/2008/9/5//R89)

To maximise collaborations further, these sites could be networked, as part of a global sociotechnical system. Lessons for real-time response can also be drawn from disaster management, and how sociotechnical systems operate to achieve real-time outcomes (Callaghan, 2016). Effectively, the development of a global sociotechnical system (or GPP to solve global societal problems) entails the democratisation of knowledge and processes, ensuring their contribution as a public good; this also entails engagement, and enablement, of different stakeholder groups (Shum et al., 2012). Shum et al. (2012, p. 109) define collective intelligence as ‘ … behaviour that is both collective and intelligent,’ collective meaning ‘groups of individual actors, including, for example, people, computational agents, and organisations,’ and intelligent meaning that ‘the collective behaviour of the group exhibits characteristics such as, for example, perception, learning, judgement, or problem solving.’ Increasing networked collaborations might be considered akin to the development of a social movement, based on open modes of knowledge creation, seeking to leverage collective intelligence.

Wikipedia is an example of a successful collaborative system which has achieved a low-cost way of attracting online participants; evidence indicates that although in the beginning a core group of elite contributors were dominant in contributions, over time common users have come to make most of the contributions (Kittur, Chi, Pendleton, Suh, & Mytkowicz, 2007). This is analogous to Malone and Klein’s (2007) notion of ‘copilots,’ where a ‘vertical’ support function is also undertaken by the crowd.

If those at the coalface of the problem (the ‘swarm’ or ‘crowd’ at work at the ‘coalface’) can be taken to be the horizontal problem-solving interface, those providing coordination and decision-making activities can be regarded as the vertical problem-solving interface. Both horizontal and vertical problem-solving therefore draw from the ‘wisdom of crowds’ effect (Surowiecki, 2004). Wikipedia reflects a ‘wisdom of crowds’ effect; the study of Wikipedia has important implications for ‘the design of novel collaborative knowledge systems’ (Kittur et al., 2007, p. 1). This type of system is itself changed over time by those that adopt it, sharing characteristics with dynamic social systems and reflecting changes in the perspectives of their underlying contributors; those that come before seem to increase the utility available in the system (Kittur et al., 2007).

This shift ‘to the novice masses’ reflects high population growth in the system, and with it the need for structure, procedure and hierarchies (Kittur et al., 2007, p. 8). Kittur et al. (2007) argue that this online social system population might reflect social stratification of broader societies. The implication of this is that certain structures might be similar across different levels of social structures. This ties in with a theme of this paper; that different ‘laws of the crowd’ might exist across different levels which can be used to maximise global problem-solving and that the field of probabilistic innovation needs to theorise around these laws in order to be able to offer insights for real-time research problem-solving.

This emerging problem-solving literature offers a vista of new and novel implications and opportunities with regard to engineering crowd-based research problem-solving systems. With regard to this particular stream of work, Malone and Klein (2007, p. 24) posit that ‘proxy democracy’ can be used on the global problem-solving site, where, ‘rather than expecting everyone to vote on all issues, users could give their voting proxies to other individuals or groups whenever they wanted to,’ allowing contributors to contribute knowledge at the level of detail they want, and individuals or groups wanting to increase their influence would need to negotiate with others and to lobby support in order to control more proxies. Organisations that post problems on the innovation mall InnoCentive.com follow the same hierarchical structure, as these problems are typically ‘smaller pieces of the sponsors’ much larger R&D programs’ and these organisations usually have sound knowledge of relevant technologies as well as user needs and functional requirements (markets), which allows them to define system configurations and coordinate collaborator work (Pistano & Vergani, 2008). Arguably, the hierarchical organisation of problem-solving work can be taken to represent the vertical axis of crowd problem-solving, and flat modes of problem-solving may represent the horizontal interface.

Flat modes are suited to projects like open-source software development, where the problems addressed are identified and defined by users, who are closest to the problem and best placed to devise and test solutions; another example of a flat mode is IBM’s microelectronics consortia, which enables contributing organisations to use developed technologies in their own product lines, with equal input (Pistano & Vergani, 2008). Under conditions of maximum collaboration, large numbers of crowd participants could be ‘spread out’ over the problem space, akin to the ‘coalface’ analogy, in a type of flat mode of engagement with different dimensions of the problem. This would take the form of a giant web-forum based on increasing the probabilistic engagement of solvers with the problem.

A large problem-solving web-based forum (for example targeted at solving global warming) could act as ‘simultaneously, a kind of Wikipedia for controversial topics, a Sims game for the future of the planet, and an electronic democracy’ and ‘if we could build it, our societal conversation about global warming could go beyond the realm of the all-too-often emotionally-driven yes/no votes about small numbers of simplified alternatives,’ facilitating ‘reasoned and evidence-based collective decision-making about highly complex issues’ (Malone & Klein, 2007, p. 25). Whereas Malone and Klein focus on the global problem of global warming, it is argued here other pressing global problems can benefit from this body of literature relating to how problems can be solved in real time using global problem-solving platforms and the inputs of large numbers of people. Managing large-scale inputs in such a system, however, assumes a subordinate system of input generation.

High volumes of input generation are another advantage offered by the innovation contest model. A typical innovation contest involves firms, termed ‘seekers’ who post innovation-related, or technical, R&D problems, for populations of independent agents, termed ‘solvers,’ to address using an open innovation process, typically with an award offered for the best solution (Terwiesch & Xu, 2008). Individuals and online communities contributing ideas in open innovation processes have already been found to be motivated by altruism, competition for status, or the self-interest typically related to being a user; however, in addition solvers can also be motivated using innovation contests, or innovation tournaments. Examples of innovation contests include the DARPA Grand Challenge for autonomous robotic vehicles, and as discussed previously, InnoCentive, an innovation platform conducting hundreds of innovation contests annually in which about 95,000 solvers around the world compete to solve problems, mostly in molecular biology and chemistry (for awards that are typically between $10,000 to $50,000) (Terwiesch & Xu, 2008).

What makes innovation contests useful to seekers is that competition between solvers is enabled, seekers only pay for successes and not failures, risk of failure shifts to solvers, wage-rate arbitrage cost advantages are enabled, and large-scale idea generation and testing is facilitated; large corporates, including Ely Lilly and Du Pont, have used InnoCentive for a growing proportion of their R&D (Terwiesch & Xu, 2008).

Some have argued that organisational success is primarily a function of the rate at which an organisation can learn, and institutional learning drawing on systems dynamics methodology can be important differentiator of organisational performance (Senge & Sterman, 1992). The success of a global knowledge creation system seeking to maximise collaborations may also be dependent on the rate at which the system can learn. The literature discussed in preceding sections is taken to represent work related to how collaborative research can leveraged to better solve research problems, or relating to ‘collaborative leverage’; what links this literature is therefore its potential contribution to the goal of maximising collaborative problem-solving.

**Fig. 1.**
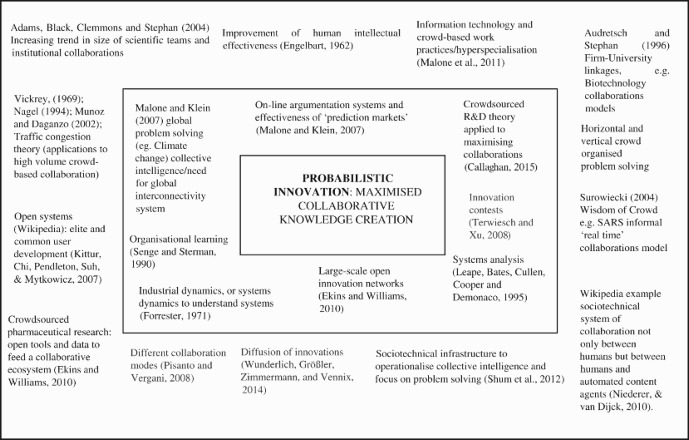
Collaborative leverage theoretical framework: constituent literatures.

Fig. 1 provides an overview of theoretical frameworks related to this ‘collaborative leverage’ theory. The theorists in the innermost frame are taken to represent those with perhaps the most salient contributions to collaborative problem-solving. Those in the outer frame are taken to offer theoretical frameworks which further support the collaborative leverage process. The identification, and potential integration, of these subordinate bodies of theory into a core body of literature to support maximised collaborative problem-solving systems is considered important, for the following reasons. *First*, it is argued technological advancements have enabled what is essentially a new paradigm in collaborative problem-solving, and that over and above accelerating trends toward collaborations (Adams et al., 2004), a proactive attempt to ‘catch’ this building wave and operationalise its potential may result in dramatic improvements in global social problem-solving.

*Second*, in the face of global social challenges such as HIV, rising chronic disease burdens, threats of microbial disease outbreaks and antibiotic resistance, seeking to radically maximise collaborative engagement between researchers and all other stakeholders may provide important scale advantages.

*Third*; arguably, the rise of the body of literature associated with probabilistic innovation heralds a new movement, spurred by social media, related to the democratisation of science and problem-solving. Dominant in this body of literature is the simple notion that increased networking and collaborations can accelerate research and problem-solving, and that a radical approach to increasing these also required scholarly attention. Increased transparency and participation by stakeholders of all stripes is perhaps already changing the power dynamics of science and its problem-solving processes, reflecting a global shift toward probabilistic innovation; this shift is perhaps a necessary condition, in that the lack of outright solutions to apocalyptic problems sooner or later will trigger the necessity as ‘mother of invention’ effect, and people will ultimately start to ‘self-organise’ and begin to develop radical and novel solutions in response to these crises. This paper has sought to do just this, and to document emergence of these ideas. Further development of this stream of literature may usefully seek to document and investigate these ideas further. It is therefore hoped this paper has in some way contributed to this project.

## 7. Conclusions

The objective of this paper was to identify and synthesise a stream of literature which offers new hope for those seeking to solve global crises with potential apocalyptic consequences, or with very large potential consequences if not solved. HIV, antiretroviral resistance and antibiotic resistance were offered as examples of these problems. A body of theory was identified and incorporated into discussion of what might be an emerging field, relating to how global systems of networked collaborations could usefully contribute to a radical new paradigm of maximised collaborative problem-solving. It was also argued that these developments reflect a deeper movement, perhaps on the scale of a social movement, toward the democratisation of science and the involvement of all affected stakeholders as problem-solving collaborators in some form or the other. These changes might herald the advent of a new era of transparency, harnessing of collective intelligence and the involvement (and legitimisation of involvement) of the ‘crowd’ in matters that affect it.
